# Effect of recent and ancient inbreeding on production and fertility traits in Canadian Holsteins

**DOI:** 10.1186/s12864-020-07031-w

**Published:** 2020-09-01

**Authors:** Bayode O. Makanjuola, Christian Maltecca, Filippo Miglior, Flavio S. Schenkel, Christine F. Baes

**Affiliations:** 1 Centre for Genomic Improvement of Livestock, Department of Animal Biosciences, University of Guelph, Guelph, ON N1G 2W1 Canada; 2 Department of Animal Science and Genetics Program, North Carolina State University, Raleigh, NC 27607 USA; 3 Institute of Genetics, Vetsuisse Faculty, University of Bern, 3001 Bern, Switzerland

**Keywords:** Inbreeding depression, Recent and ancient inbreeding, Pedigree and genomic inbreeding

## Abstract

**Background:**

Phenotypic performances of livestock animals decline with increasing levels of inbreeding, however, the noticeable decline known as inbreeding depression, may not be due only to the total level of inbreeding, but rather could be distinctly associated with more recent or more ancient inbreeding. Therefore, splitting inbreeding into different age classes could help in assessing detrimental effects of different ages of inbreeding. Hence, this study sought to investigate the effect of recent and ancient inbreeding on production and fertility traits in Canadian Holstein cattle with both pedigree and genomic records. Furthermore, inbreeding coefficients were estimated using traditional pedigree measure (*F*_*PED*_) and genomic measures using segment based (*F*_*ROH*_) and marker-by-marker (*F*_*GRM*_) based approaches.

**Results:**

Inbreeding depression was found for all production and most fertility traits, for example, every 1% increase in *F*_*PED*_, *F*_*ROH*_ and *F*_*GRM*_ was observed to cause a − 44.71, − 40.48 and − 48.72 kg reduction in 305-day milk yield (MY), respectively. Similarly, an extension in first service to conception (FSTC) of 0.29, 0.24 and 0.31 day in heifers was found for every 1% increase in *F*_*PED*_, *F*_*ROH*_ and *F*_*GRM*_, respectively. Fertility traits that did not show significant depression were observed to move in an unfavorable direction over time. Splitting both pedigree and genomic inbreeding into age classes resulted in recent age classes showing more detrimental inbreeding effects, while more distant age classes caused more favorable effects. For example, a − 1.56 kg loss in 305-day protein yield (PY) was observed for every 1% increase in the most recent pedigree age class, whereas a 1.33 kg gain was found per 1% increase in the most distant pedigree age class.

**Conclusions:**

Inbreeding depression was observed for production and fertility traits. In general, recent inbreeding had unfavorable effects, while ancestral inbreeding had favorable effects. Given that more negative effects were estimated from recent inbreeding when compared to ancient inbreeding suggests that recent inbreeding should be the primary focus of selection programs. Also, further work to identify specific recent homozygous regions negatively associated with phenotypic traits could be investigated.

## Background

Over the past decade, Canadian Holstein cattle populations have experienced an increase in the annual rate of inbreeding from 0.08 to 0.23%, which were observed from 2000 to 2010 and 2010–2018, respectively [1]. Recently, Makanjuola et al. [2] estimated the effective population size for North American Holsteins to range from 43 to 66 using genotyped animals. The small effective population size and the increasing rate of inbreeding could result in a phenomenon known as inbreeding depression. Inbreeding depression is the noticeable decline in the phenotypic mean of economically important traits within a given population [3]. This decline is often attributable to decreasing heterozygosity and increasing recessive homozygosity resulting from inbreeding and random genetic drift. The underlying genetic mechanism of inbreeding depression has been categorized into three hypotheses, which includes partial dominance, over-dominance and epistasis hypotheses. In the partial dominance hypothesis, depression is observed when inbreeding exposes deleterious recessive alleles that were previously hidden in heterozygous state [4]. In the over-dominance hypothesis, over-dominance contributes to inbreeding depression by reducing heterozygous genotypes that show superiority over the two homozygous genotypes [5]. In the epistasis hypothesis, depression could result when inbreeding reduces the combination of favorable heterozygous genotypes across multiple loci [6]. From these hypotheses, partial dominance has been widely reported to account for most of the observed inbreeding depression [4, 7, 8].

Before the availability and popularity of genomic data, estimation of inbreeding depression was predominantly done by calculating inbreeding coefficients from pedigree data and regressing any trait of economic interest on the inbreeding coefficients [9, 10]. More recently, genomic inbreeding estimates are being used to assess inbreeding depression [11, 12]. Genomic inbreeding coefficients have been shown to be closer to true inbreeding estimates [13]. This could be because Mendelian sampling variation are better accounted for by genomic data [14] and genomic data are independent of pedigree depth and completeness [15]. Different methods have been used for estimating genomic inbreeding. Genomic inbreeding could be estimated by subtracting one from the diagonal of the genomic relationship matrix [16, 17]. Alternatively, McQuillan et al. [18] proposed the estimation of genomic inbreeding from unbroken stretches of homozygous segments, which are referred to as runs of homozygosity (ROH).

Ideally, the aim of the genetic selection being practised in livestock species is to increase the frequency of favorable alleles, thus, increasing the level of homozygosity. In essence, inbreeding could result in the depression or enhancement of any trait of economic interest, therefore not all inbreeding is detrimental. Inbreeding increases the expression of deleterious recessive alleles, which are naturally or artificially selected against in a process called genetic purging [19, 20]. With the theory of genetic purging, inbreeding coefficients could be partitioned into ancient and recent inbreeding. Ancient inbreeding is inbreeding that occurred from a distant common ancestor and, as such, is expected to show less unfavorable effect due to genetic purging, whereas recent inbreeding is inbreeding that arose from a most recent common ancestor and hence is expected to exhibit larger unfavorable effects [21]. For example, Doekes et al. [22] reported a 2.42 kg decline in fat yield (FY) per 1% increase in new inbreeding and conversely, an increase of 0.03 kg for ancient inbreeding.

The partitioning of inbreeding into recent and ancient inbreeding can be examined with pedigree and genomic data. For pedigree data, recent inbreeding can be estimated by tracing the pedigree back relatively few generations to the common ancestor, while ancient inbreeding traces back the pedigree to a more distant common ancestor [23]. In addition, classical inbreeding coefficients could be divided into new and ancestral inbreeding based on whether alleles carried by an individual have previously occurred in an identity-by-descent (IBD) state in an ancestor or are occurring for the first time in an IBD state [24, 25]. For genomic data, recent and ancient inbreeding can be separated by allocating the length of the ROH into different classes. Over time, recombination tends to breakdown long chromosomal segments, thus longer ROH could suggest recent inbreeding due to lack of time for recombination, and shorter lengths indicate ancient inbreeding [26]. Inferring the age of inbreeding from the length of ROH segment is an expectation that follows an exponential distribution with a mean of 100/2 *g* centiMorgans (cM), where *g* is the number of generations to a common ancestor [27].

The objectives of this study were to 1) estimate the effect of inbreeding on production and fertility traits in Canadian Holsteins using pedigree and genomic information; 2) assess the effect of recent and ancient inbreeding on production and fertility traits in Canadian Holsteins.

## Results

### Phenotypic description, heritability and inbreeding coefficients

The basic descriptive statistics of the phenotypic data are presented in Table 1. This include the total number of records for each trait evaluated with their respective observations. Moderate heritability estimates of 0.26, 0.23 and 0.22 were obtained for MY, FY and PY, respectively (Table 2). As expected, heritability estimates were low for fertility traits and ranged from 0.01 to 0.07 (Table 2).

**Table 1:** Descriptive statistics of the evaluated traits, including number of records, mean, standard deviation, minimum and maximum number of observations

Traits^a^	N	Mean	SD	Min	Max
MY (kg)	21,194	9074	1732.22	1140	17,542
FY (kg)	21,194	362	73.85	39	919
PY (kg)	21,194	295.50	54.58	39	603
AFS_H (day)	33,610	449.20	49.16	274	639
NS_H	33,610	1.59	0.93	1	7
NRR_H	33,610	0.69	0.46	0	1
FSTC_H (day)	33,610	19.32	33.37	0	205
CTFS_C (day)	19,338	79.88	24.11	20	243
NS_C	19,338	1.98	1.25	1	10
NRR_C	19,338	0.55	0.50	0	1
FSTC_C (day)	19,338	32.75	43.80	0	206

^a^ *MY* milk yield, *FY* fat yield, *PY* protein yield, *AFS_H* age at first service for heifers, *NS_H* number of service for heifers, *NRR_H* 56-day non-return rate for heifers, *FSTC_H* first service to conception for heifers, *CTFS_C* conception to first service for cows, *NS_C* number of service for cows, *NRR_C* 56-day non-return rate for cows, *FSTC_C* first service to conception for cows

**Table 2:** Estimates of phenotypic variance and ratios with respect to phenotypic variance for additive genetic (h^2^), herd within region-year-season (hrys^2^), service sire by year of insemination (ss^2^), artificial insemination technician (tech^2^), and residual (e^2^) variances (standard errors in parentheses)

Traits^a^	Phenotypic variance	h^2^ (%)	hrys^2^ (%)	tech^2^ (%)	ss^2^ (%)	e^2^ (%)
MY (kg)	2,740,757	26.02 (1.89)	28.17 (0.90)			45.81 (1.79)
FY (kg)	4889.50	22.57 (1.77)	31.98 (0.90)			45.45 (1.68)
PY (kg)	2663.70	21.69 (1.67)	33.35 (0.90)			44.96 (1.60)
AFS_H (day)	2410.50	4.25 (0.67)	60.24 (0.56)			35.51 (0.71)
NS_H	0.87	1.96 (0.42)	6.81 (0.54)			91.23 (0.67)
NRR_H	0.21	1.31 (0.25)	2.78 (0.47)	1.44 (0.03)	0.56 (0.16)	93.91 (0.58)
FSTC_H (day)	1118.60	2.12 (0.47)	6.20 (0.55)			91.68 (0.70)
CTFS_C (day)	594.27	6.59 (1.13)	39.90 (1.01)			53.51 (1.32)
NS_C	1.65	4.21 (0.85)	2.78 (0.46)			93.01 (1.08)
NRR_C	0.25	3.07 (0.58)	2.33 (0.59)	0.64 (0.33)	0.03 (0.12)	93.93 (0.81)
FSTC_C (day)	1920.90	3.19 (0.93)	3.18 (0.69)			93.63 (1.13)

^a^ *MY* milk yield, *FY* fat yield, *PY* protein yield, *AFS_H* age at first service for heifers, *NS_H* number of service for heifers, *NRR_H* 56-day non-return rate for heifers, *FSTC_H* first service to conception for heifers, *CTFS_C* conception to first service for cows, *NS_C* number of service for cows, *NRR_C* 56-day non-return rate for cows, *FSTC_C* first service to conception for cows

**Fig. 1:**
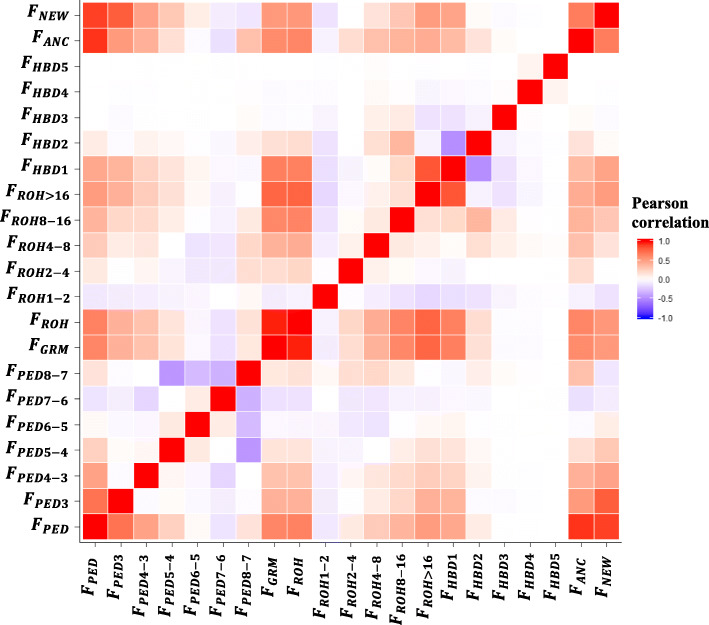
Pearson correlations of the different inbreeding measures represented by a heat map. *F*_*PED*_ - classical pedigree inbreeding; *F*_*PED*3_ - age of pedigree inbreeding based on the first three generations; *F*_*PED*4 − 3_ - age of pedigree inbreeding based on the differences between four and three generations; *F*_*PED*5 − 4_ - age of pedigree inbreeding based on the differences between five and four generations; *F*_*PED*6 − 5_ - age of pedigree inbreeding based on the differences between six and five generations; *F*_*PED*7 − 6_ - age of pedigree inbreeding based on the differences between seven and six generations; *F*_*PED*8 − 7_ - age of pedigree inbreeding based on the differences between eight and seven generations; *F*_*ROH*_ - classical genomic inbreeding based on ROH; *F*_*GRM*_ - classical genomic inbreeding based on GRM; (*F*_*ROH*1 − 2_, *F*_*ROH*2 − 4_, *F*_*ROH*4 − 8_, *F*_*ROH*8 − 16_ and *F*_*ROH* > 16_) - age of genomic inbreeding based on the length of ROH (1–2, 2–4, 4–6, 8–16 and > 16 Mb, respectively); (*F*_*HBD*1_, *F*_*HBD*2_, *F*_*HBD*3_, *F*_*HBD*4_ and *F*_*HBD*5_) - age of genomic inbreeding based on model-based approach; *F*_*ANC*_ and *F*_*NEW*_ - Kalinowski’s ancestral and new inbreeding

The correlation coefficients of all estimated inbreeding coefficients are depicted in Fig. 1. The correlation coefficients between classical pedigree inbreeding and classical genomic inbreeding were moderately high at 0.63 for *F*_*PED*_ and *F*_*ROH*_ and 0.61 for *F*_*PED*_ and *F*_*GRM*_. A correlation of 0.97 was estimated for *F*_*ROH*_ and *F*_*GRM*_ (Fig. 1). More interestingly were the correlations between the classical inbreeding estimates and the different ages of inbreeding measures, where *F*_*PED*_, *F*_*ROH*_ and *F*_*GRM*_ had moderately positive correlations with recent generations and dropped to low and negative values as the generation became more ancient. For example, *F*_*PED*_, *F*_*ROH*_ and *F*_*GRM*_ had a correlation of 0.70, 0.40 and 0.40 with the most recent pedigree age of inbreeding (*F*_*PED*3_), respectively, whereas, a correlation with a more distant pedigree age of inbreeding (*F*_*PED*7 − 6_) was equal to − 0.12, − 0.13 and − 0.12, respectively. Similarly, correlations between *F*_*PED*_, *F*_*ROH*_ and *F*_*GRM*_ with *F*_*ROH* > 16_ were estimated to be 0.51, 0.77 and 0.76, respectively, in contrast to − 0.10, − 0.06 and − 0.08 for *F*_*ROH*2 − 4_, respectively. For the model-based age of genomic inbreeding, the correlations ranged from 0.44 to 0.65 for the most recent age and − 0.01 to 0.00 for the most distant age of genomic inbreeding with the classical inbreeding measures. The movement in different directions of the correlations with the different classes of the age of inbreeding was notable, with correlations ranging from − 0.45 to 0.11 for the pedigree classes, − 0.17 to 0.17 for the ROH classes and − 0.48 to 0.06 for the model-based classes (Fig. 1).

### Effect of classical inbreeding on phenotypic traits

Statistically significant inbreeding depression (*P* < 0.01) was observed for all production traits based on *F*_*PED*_, *F*_*ROH*_ and *F*_*GRM*_ (Table 3). For every 1% increase in inbreeding coefficients based on *F*_*PED*_, *F*_*ROH*_ and *F*_*GRM*_, a corresponding reduction of 44.71, 40.48 and 48.72 kg was estimated, respectively, representing 0.49, 0.45 and 0.54% of the phenotypic means for the traits. Likewise, the effect of inbreeding was noticeable for fertility traits with heifers having a statistically significant (*P* < 0.05) increase of 0.29, 0.24 and 0.31 days in FSTC for every 1% increase in inbreeding coefficients based on *F*_*PED*_, *F*_*ROH*_ and *F*_*GRM*_, respectively, which represents 1.50, 1.24 and 1.60% of the phenotypic means. Despite the statistically non-significant effects (*P* < 0.44) for cows, a 1% increase in inbreeding coefficients based on *F*_*PED*_, *F*_*ROH*_ and *F*_*GRM*_ resulted in an extension of 0.16, 0.19 and 0.19 days, respectively, for FSTC. In addition, both heifers and cows showed genomic inbreeding depression for number of services (NS). For instance, a 1% increase in *F*_*ROH*_ resulted in a 0.78 and 0.83 chance of getting re-inseminated after the first insemination for heifers and cows, respectively. An inbreeding depression of 0.96 was observed in NS (*P* < 0.01) for a 1% increase in *F*_*PED*_ for heifers, while a statistically non-significant (*P* < 0.51) effect of 0.45 was observed for a 1% increase in *F*_*PED*_ for cows. To further support the effect of inbreeding, differences in the phenotypic means of animals with low inbreeding levels (5th percentile) and high inbreeding levels (95th percentile) were estimated and are presented in Table 4. On average, lowly inbred animals produced 144.69, 342.85 and 435.77 kg more milk than highly inbred animals when estimates were based on *F*_*PED*_, *F*_*ROH*_ and *F*_*GRM*_, respectively. In a similar fashion, animals with low inbreeding coefficients had 3.26, 5.04 and 7.10 kg more FY based on *F*_*PED*_, *F*_*ROH*_ and *F*_*GRM*_, respectively, when compared to animals with high inbreeding coefficients. For fertility traits, heifers with high inbreeding levels had on average 6.40, 6.71 and 5.39 more days to age at first insemination (AFS) based on *F*_*PED*_, *F*_*ROH*_ and *F*_*GRM*_, respectively. Likewise, 2.83, 4.12 and 3.87 less days for FSTC was estimated based on *F*_*PED*_, *F*_*ROH*_ and *F*_*GRM*_, respectively, for heifers with low inbreeding compared to heifers with high inbreeding. For cows, a more evident increase in NS of 3.56, 1.04 and 1.82% based on *F*_*PED*_, *F*_*ROH*_ and *F*_*GRM*_, respectively, was estimated for highly inbred cows in comparison to lowly inbred cows.

**Table 3:** Estimates of inbreeding depression on production and fertility traits per 1% increase in classical inbreeding and their standard errors

Traits^**a**^	***F***_***PED***_^**b**^		***F***_***ROH***_^**b**^		***F***_***GRM***_^**b**^	
	Estimates	SE	Estimates	SE	Estimates	SE
MY (kg)	−44.71^***^	6.47	−40.48^***^	3.80	−48.72^***^	4.49
FY (kg)	−1.65^***^	0.27	−1.40^***^	0.16	−1.75^***^	0.19
PY (kg)	−1.38^***^	0.20	−1.26^***^	0.12	−1.52^***^	0.14
AFS_H (day)	0.44^***^	0.14	0.33^***^	0.08	0.35^***^	0.09
NS_H^c^	0.96^***^	0.32	0.78^***^	0.19	0.99^***^	0.22
NRR_H^c^	−0.01	0.16	−0.14	0.09	−0.19^*^	0.11
FSTC_H (day)	0.29^**^	0.12	0.24^***^	0.07	0.31^***^	0.08
CTFS_C (day)	0.07	0.09	0.04	0.05	0.02	0.06
NS_C^c^	0.45	0.51	0.83^***^	0.29	0.70^**^	0.34
NRR_C^c^	−0.29	0.23	−0.34^**^	0.14	−0.33^**^	0.16
FSTC_C (day)	0.16	0.21	0.19	0.12	0.19	0.14

^***^P < 0.01; ^**^P < 0.05; ^*^P < 0.1

^a^*MY* milk yield, *FY* fat yield, *PY* protein yield, *AFS_H* age at first service for heifers, *NS_H* number of service for heifers, *NRR_H* 56-day non-return rate for heifers, *FSTC_H* first service to conception for heifers, *CTFS_C* conception to first service for cows, *NS_C* number of service for cows, *NRR_C* 56-day non-return rate for cows, *FSTC_C* first service to conception for cows

^b^*F*_*PED*_ = pedigree inbreeding; *F*_*ROH*_ = segment-based genomic inbreeding; *F*_*GRM*_ = marker-by-marker-based genomic inbreeding

^c^Trait estimates and SE were multiplied by 100

**Table 4:** Estimates of inbreeding depression for all significant traits, expressed as the difference (Diff) in predicted phenotype between lowly inbred (5% percentile) and highly inbred (95% percentile) from the mean for *F*_*PED*_, *F*_*ROH*_ and *F*_*GRM*_

Traits^**a**^	***F***_***PED***_^**b**^			***F***_***ROH***_^**b**^			***F***_***GRM***_^**b**^		
	Low	High	Diff	Low	High	Diff	Low	High	Diff
MY (kg)	9031.89	8887.20	144.69	9152.62	8809.77	342.85	9233.88	8798.11	435.77
FY (kg)	357.35	354.09	3.26	359.29	354.25	5.04	362.09	355.00	7.10
PY (kg)	294.74	292.17	2.57	296.79	289.71	7.08	299.54	289.33	10.21
AFS (day)	450.51	456.91	−6.40	451.04	457.75	−6.71	451.13	456.52	−5.39
NS^c^	37.03	39.48	−2.45	35.34	40.21	−4.87	35.75	40.58	−4.83
NRR^d^	70.27	69.55	0.72	70.61	68.05	2.56	70.49	67.68	2.81
FSTC (day)	19.47	22.30	−2.83	18.22	22.34	−4.12	18.43	22.30	−3.87
NS^c^ (%)	52.43	55.99	−3.56	53.67	54.71	−1.04	53.30	55.12	−1.82
NRR^d^ (%)	54.50	52.58	1.92	54.60	53.46	1.14	54.43	53.36	1.07

^a^*MY* milk yield, *FY* fat yield, *PY* protein yield, *AFS_H* age at first service for heifers, *NS_H* number of service for heifers, *NRR_H* 56-day non-return rate for heifers, *FSTC_H* first service to conception for heifers, *CTFS_C* conception to first service for cows, *NS_C* number of service for cows, *NRR_C* 56-day non-return rate for cows, *FSTC_C* first service to conception for cows

^b^*F*_*PED*_ = pedigree inbreeding; *F*_*ROH*_ = segment-based genomic inbreeding; *F*_*GRM*_ = marker-by-marker-based genomic inbreeding

^c^Incidence of more than one service after first

^d^Incidence of no subsequent service between 15 and 56 days following the first service

### Effect of age of inbreeding on phenotypic traits

**Fig. 2:**
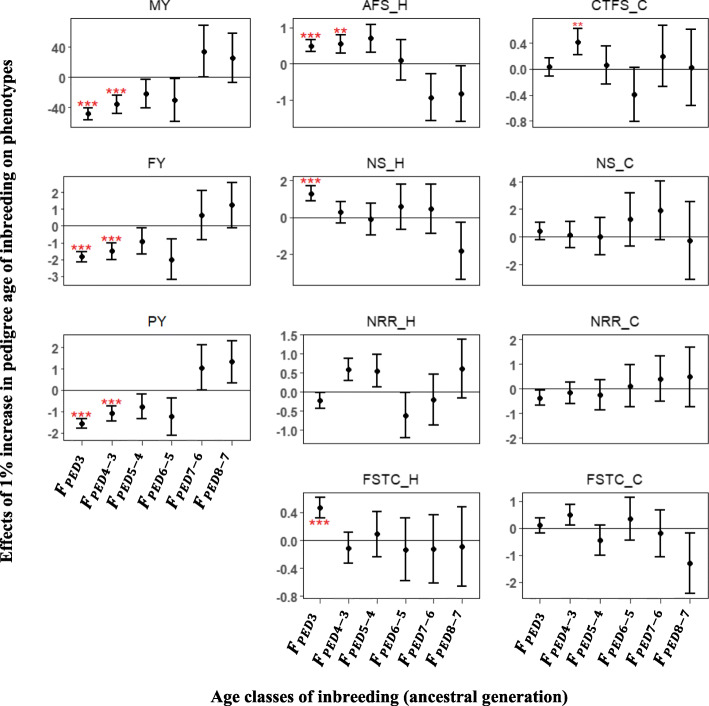
Effect of a 1% increase in pedigree age inbreeding estimated on phenotypes. Error bars represent one standard error and stars indicate significance level (^***^P < 0.01; ^**^P < 0.05; ^*^P < 0.1). MY- milk yield; FY- fat yield; PY- protein yield; AFS_H - age at first service for heifers; NS_H - number of service for heifers; NRR_H - 56-day non-return rate for heifers; FSTC_H-first service to conception for heifers; CTFS_C - conception to first service for cows; NS_C - number of service for cows; NRR_C - 56-day non-return rate for cows; FSTC_C - first service to conception for cows

Splitting the pedigree inbreeding coefficients into different age (generation) classes showed varying effects on phenotypes. Interestingly, inbreeding occurring within the most recent five generations resulted in unfavorable and statistically significant depressing effects on phenotypic traits, however, more distant generations showed favorable, but a statistically non-significant effects on phenotypic traits (Fig. 2). A 1% increase in the inbreeding coefficients obtained from *F*_*PED*3_, *F*_*PED*4 − 3_ and *F*_*PED*5 − 4_ caused a reduction of 1.56, 1.10 and 0.77 kg in PY, respectively. Whereas a 1% increase in *F*_*PED*7 − 6_ and *F*_*PED*8 − 7_ resulted in a corresponding 1.06 and 1.33 increase in PY, respectively. Similarly for fertility traits, AFS increased by 0.50, 0.55 and 0.70 days in heifers for every 1% increase in *F*_*PED*3_, *F*_*PED*4 − 3_ and *F*_*PED*5 − 4_, respectively, and conversely reduced by 0.93 and 0.84 days for a 1% increase in *F*_*PED*7 − 6_ and *F*_*PED*8 − 7_, respectively. For cows, a similar pattern was observed with recent generations having more negative effects and remote generations showing more positive effects. However, all estimated effects were statistically non-significant with the exception of days from calving to first insemination (CTFS), which showed a 0.42 increase in days for a 1% increase in *F*_*PED*4 − 3_ (*P <* 0.05).

**Fig. 3:**
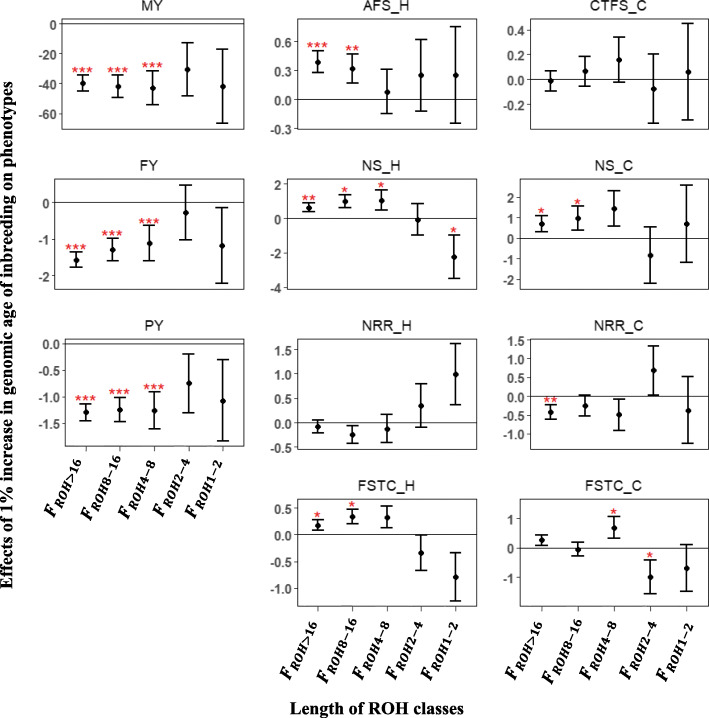
Effect of a 1% increase in genomic age inbreeding estimated using the sliding window approach on phenotypes. Error bars represent one standard error and stars indicate significance level (^***^P < 0.01; ^**^P < 0.05; ^*^P < 0.1). MY- milk yield; FY- fat yield; PY- protein yield; AFS_H - age at first service for heifers; NS_H - number of service for heifers; NRR_H - 56-day non-return rate for heifers; FSTC_H-first service to conception for heifers; CTFS_C - conception to first service for cows; NS_C - number of service for cows; NRR_C - 56-day non-return rate for cows; FSTC_C - first service to conception for cows

ROH was split into age classes with longer ROH indicating more recent inbreeding and shorter ROH suggesting more remote inbreeding. Although the effect of all ROH classes were unfavorable for production traits, only ROH classes with segments longer than 4 Mb were significant at *P* < 0.05 (Fig. 3). For example, a 1% increase in *F*_*ROH*4 − 8_, *F*_*ROH*8 − 16_ and *F*_*ROH* > 16_ led to a 1.12, 1.29 and 1.57 kg reduction in FY, respectively. For fertility traits in heifers, inbreeding effect on 56-day non-return rate (NRR) was not statistically significant for all ROH classes, however, shorter segments (ROH < 4 Mb) showed favorable effects while longer segments (ROH > 4 Mb) had unfavorable effects. For AFS in heifers, unfavorable inbreeding effects for all classes of ROH were observed, but only ROH > 8 Mb showed statistical significance (*P* < 0.05). Additionally, a statistically significant and unfavorable effect of 0.62 and 0.96 was obtained for a 1% increase in *F*_*ROH*8 − 16_ and *F*_*ROH* > 16_ for NS, respectively, whereas a statistically non-significant, but favorable effect of − 2.26 and − 0.07 was obtained for, *F*_*ROH*1 − 2_ and *F*_*ROH*2 − 4_, respectively. For fertility traits in cows, only an unfavorable and statistically significant (*P* < 0.05) effect on NRR for ROH >16 Mb was observed. In general, cow traits follow a similar pattern with shorter segments tending to have favorable effects, while longer segments tending to be unfavorable.

**Fig. 4:**
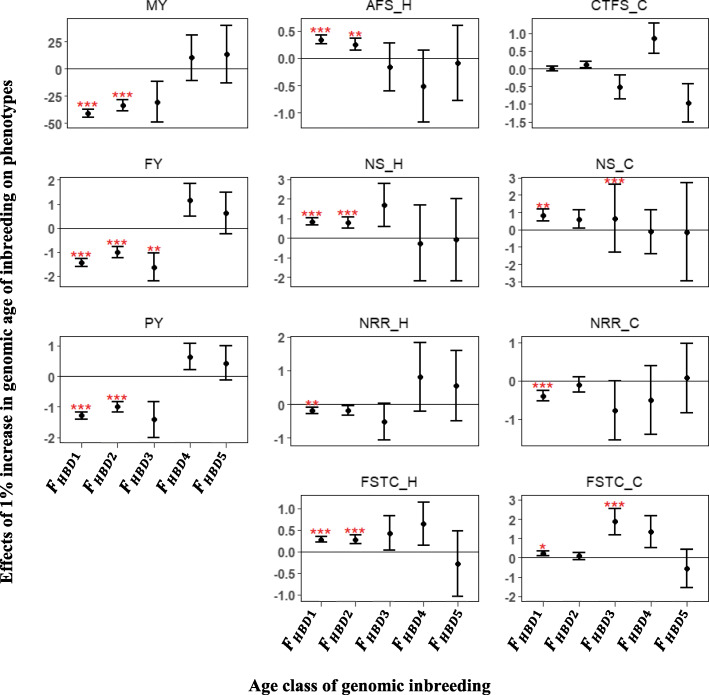
Effect of a 1% increase in genomic age inbreeding estimated using the model-based approach on phenotypes. Error bars represent one standard error and stars indicate significance level (^***^P < 0.01; ^**^P < 0.05; ^*^P < 0.1). MY- milk yield; FY- fat yield; PY- protein yield; AFS_H - age at first service for heifers; NS_H - number of service for heifers; NRR_H - 56-day non-return rate for heifers; FSTC_H-first service to conception for heifers; CTFS_C - conception to first service for cows; NS_C - number of service for cows; NRR_C - 56-day non-return rate for cows; FSTC_C - first service to conception for cows

The age of inbreeding estimated using the model-based approach provided varying effects on phenotypes. Based on this approach, more recent age of inbreeding had statistically significant and unfavorable effects on production traits and more distant ages had statistically non-significant and favorable inbreeding effects (Fig. 4). A 1% increase in *F*_*HBD*1_, *F*_*HBD*2_ and *F*_*HBD*3_ corresponded to a − 40.79, − 33.76 and − 30.53 kg loss in MY, respectively. In contrast, a 1% increase in *F*_*HBD*4_ and *F*_*HBD*5_ was related to 10.06 and 15.65 kg gain in MY, respectively. For fertility traits, a 1% increase in *F*_*HBD*1_ and *F*_*HBD*2_ in heifers prolonged FSTC by 0.28 and 0.28 days, respectively. Conversely, a 1% increase in *F*_*HBD*5_ reduced FSTC by 0.42 days, although, this was statistically non-significant. In addition, a statistically significant increase of 0.83 in NS for cows with a 1% increase in *F*_*HBD*1_ and a statistically non-significant decrease of 0.22 for every 1% increase in *F*_*HBD*4_ in NS in cows was estimated.

### Effect of new and ancestral inbreeding on phenotypic traits

**Fig. 5:**
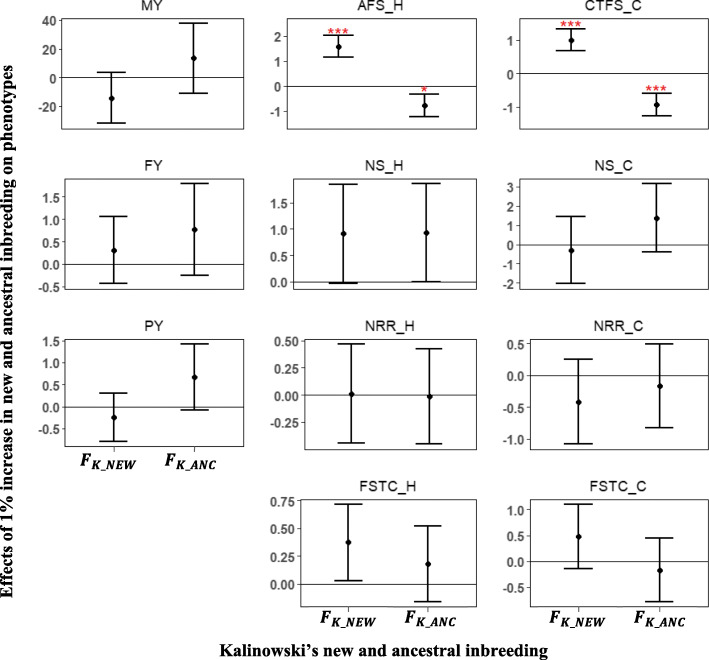
Effect of a 1% increase in new and ancestral inbreeding estimated using kalinowski’s method on phenotypes. Error bars represent one standard error and stars indicate significance level (^***^P < 0.01; ^**^P < 0.05; ^*^P < 0.1). MY- milk yield; FY- fat yield; PY- protein yield; AFS_H - age at first service for heifers; NS_H - number of service for heifers; NRR_H - 56-day non-return rate for heifers; FSTC_H-first service to conception for heifers; CTFS_C - conception to first service for cows; NS_C - number of service for cows; NRR_C - 56-day non-return rate for cows; FSTC_C - first service to conception for cows

The partitioning of the classical inbreeding into new and ancestral inbreeding as proposed by Kalinowski et al. [24] provided insight into how recent inbreeding affects phenotypes. For production traits, no significant effect was obtained with *F*_*k* _ *NEW*_ and *F*_*k* _ *ANC*_ (Fig. 5). Nevertheless, *F*_*k* _ *NEW*_ showed unfavorable effects while *F*_*k* _ *ANC*_ tended towards more favorable effects. A 1% increase in *F*_*k* _ *NEW*_ resulted in a − 14.21 and − 0.24 kg loss in MY and PY, respectively. On the other hand, a 1% increase in *F*_*k* _ *ANC*_ caused a 13.35 and 0.67 kg increase in MY and PY, respectively. FY showed a favorable effect of 0.31 and 0.77 kg for both *F*_*k* _ *NEW*_ and *F*_*k* _ *ANC*_ per 1% increase, however, *F*_*k* _ *NEW*_ was less favorable than *F*_*k* _ *ANC*_. Similarly, for both heifer and cow traits, *F*_*k* _ *NEW*_ had a statistically non-significant but unfavorable effect, while *F*_*k* _ *ANC*_ had a statistically non-significant and favorable effects on phenotypes. For fertility traits, only AFS and CTFS showed statistically significant depressing effects, with an increase of 1.58 and 1.00 days, respectively, per 1% increase in *F*_*k* _ *NEW*_. A 1% increase in *F*_*k* _ *ANC*_ corresponded to a − 0.77 and − 0.94 days in AFS and CTFS, respectively.

## Discussion

This study sought to investigate the overall effect of classical inbreeding, different age classes of inbreeding and ancestral inbreeding on production and fertility traits using both pedigree and genomic measures. The accuracy of pedigree inbreeding estimates are largely dependent on the completeness and depth of the pedigree recording [28, 29]. Therefore, only animals with a complete generation equivalence (CGE) of 10 or more and at least 0.90 pedigree completeness index (PCI) were retained for further analyses, to prevent the underestimation of inbreeding coefficients and inbreeding depression. In the present study as well as previous studies, *F*_*PED*_ was moderately correlated with *F*_*ROH*_ and *F*_*GRM*_. In Dutch Holstein-Friesian cows, Doekes et al. [22] reported a correlation of 0.66 between *F*_*PED*_ and *F*_*ROH*_ and a correlation 0.61 between *F*_*PED*_ and *F*_*GRM*_. Similarly for Finnish Ayrshire cows, a correlation that ranged from 0.55 to 0.59 was reported by Martikainen et al. [30]. The correlations from this study and those other authors are slightly lower than those reported for bulls, which ranged from 0.67–0.87 for Australian Holstein bulls [31] and 0.70–0.75 for bulls from multiple breeds of cattle [32]. This could imply that bulls generally have more accurate pedigree records in comparison to cows.

### Classical inbreeding depression

As with other studies, a 1% increase in pedigree inbreeding has been shown to have a significantly negative effect on production traits [33–36], which ranged from − 19 to − 173 kg for MY and are in line with those reported in this study. The pedigree inbreeding effects estimated in the present study represented 0.49, 0.46 and 0.47% of the phenotypic means of MY, FY and PY, respectively. These results are in accordance with the 0.47 0.45 and 0.45% reported by Doekes et al. [22] for MY, FY and PY, respectively. For fertility traits, varying effects of pedigree inbreeding was observed. For all cow traits in the present study, there was no significant effect of pedigree inbreeding and this corroborates the results of Martikainen et al. [30], as they also found no significant association of pedigree inbreeding with fertility traits. However for heifers, an extension of 0.44 days per 1% increase in inbreeding was observed for AFS, which is similar to the 0.55 days per 1% reported by Smith et al. [9] for age at first calving (AFC; a similar trait to AFS). With genomic inbreeding measures, Bjelland et al. [37] reported a reduction of − 20 and − 47 kg per 1% increase in 205-day MY using *F*_*ROH*_ and *F*_*GRM*_, respectively. These results are in line with those reported in this study, however, the higher effect reported for *F*_*ROH*_ in the present study may be attributable to the differences in parameters used in detecting ROH. Furthermore, the effect of *F*_*ROH*_ and *F*_*GRM*_ was found to prolong interval from first to last insemination (IFL) by 0.27 and 0.42 days, respectively [22]. This trait is similar to FSTC used in this study, which was increased by 0.24 and 0.31 days per 1% increase in *F*_*ROH*_ and *F*_*GRM*_, respectively. Using genomic inbreeding, Martikainen et al. [30] also found deteriorating effect on NRR and IFL, which are supported in this study. Genomic inbreeding accounted for more phenotypic mean differences between lowly and highly inbred animals when compared to pedigree inbreeding. For example, the differences between lowly and highly inbred animals for MY was estimated to be 342.85 and 435.77 kg using *F*_*ROH*_ and *F*_*GRM*_, respectively. This is in line with the 301 and 315 kg difference between lowly and highly inbred cows reported by Doekes et al. [22] and 161 and 438 kg reported by Bjelland et al. [37] using *F*_*ROH*_ and *F*_*GRM*_, respectively. Despite *F*_*PED*_ having a higher estimated effect of inbreeding on phenotypes compared to *F*_*ROH*_, *F*_*ROH*_ accounted for a larger difference in phenotypic means between lowly and highly inbred animals. These results are similar to those reported in Doekes et al. [22] and is most likely attributable to the wider distribution of *F*_*ROH*_ over *F*_*PED*_.

### Age classes of inbreeding depression

Few studies have investigated the effect of pedigree and genomic inbreeding age classes on phenotypes [21, 23]. These age classes are supposed to represent how recent or ancient the observed inbreeding is to a common ancestor. In this study, it was hypothesized that recent inbreeding would be more detrimental than ancient inbreeding. Pedigree inbreeding traced back to ancestors in the third and fourth generation had significant negative effects on MY, FY and PY (Fig. 2). Consistent with these results, Silió et al. [23] reported a − 0.06 kg and 2.11 kg loss in daily growth rate and weight at 90 days, respectively, when pedigree was traced back to the fifth generation (*F*_*PED*5_). In addition, Doekes et al. [22] reported favorable, but non-significant, effects of *F*_*PED*7 − 6_ on MY, FY, PY, IFL and calving interval (CI). These findings are in line with the favorable, but non-significant effects of *F*_*PED*7 − 6_ and *F*_*PED*8 − 7_ on production traits, AFS and FSTC in the present study. The consistency between these studies suggest that recent inbreeding is more detrimental than ancient inbreeding. Previous researchers have found effects of different ROH length classes on phenotypes [34, 38]. In US and Australian Jersey, Howard et al. [38] observed significant inbreeding depression based on ROH with at least 4 Mb on MY, FY and PY. Likewise, for Australian Holsteins, Pryce et al. [34] found that ROH longer than 3.5 Mb exhibited more significant depression on 305-day MY when compared to shorter ROH. These results are in accordance with the present study, in which significant inbreeding depression was detected for ROH > 4 Mb and non-significant, but unfavorable inbreeding effects was observed for ROH < 4 Mb on MY, FY and PY. A similar pattern was observed for heifer fertility traits (AFS, NS, NRR and FSTC), with longer ROH showing unfavorable and significant effects and shorter ROH having favorable, but non-significant effects. Conversely, ROH > 2 Mb were found to have a more significant effect on total number of spermatozoa than ROH > 4 Mb [21]. Generally, inconsistent conclusions have been reported in the literature, with either shorter ROH carrying more deleterious alleles or longer ROH harbouring more deleterious alleles [39, 40]. In agreement with those studies, unfavorable effects were identified for both short and long ROH in the present study. The use of a deterministic approach (sliding window) in identifying ROH assumes a uniform recombination rate across the genome, however, recombination rate has been reported to vary across the genome [41]. In an attempt to circumvent this limitation, IBD regions were identified using the model-based approach [27, 42]. To our knowledge, this was the first study to investigate the effect of genomic age of inbreeding on phenotypic traits using the model-based approach. The results from this approach were similar to those reported for pedigree age of inbreeding. For recent age classes, significant inbreeding effect was found for MY, FY, PY and heifer traits (AFS, NS, NRR and FSTC). In contrast, remote age classes were favorable, but their effects were non-significant. According to Druet and Gautier [27], the model-based approach allows for the detection of the age when the inbreeding occurred, hence this support the premise that recent inbreeding are more deleterious than ancient inbreeding.

### Impact of new and ancestral inbreeding on phenotypes

Some studies have evaluated the effect of new and ancestral inbreeding on phenotypic traits [22, 43, 44]. Those authors found more evidence of large inbreeding depression resulting from new inbreeding than ancestral inbreeding, therefore, postulating that purging might have helped in removing deleterious alleles from the population. In mice, Hinrichs et al. [45] estimated an inbreeding depression that ranged from − 11.53 to − 0.74 per unit increase in *F*_*NEW*_ and − 5.52 to 15.51 per unit increase in *F*_*ANC*_ for the number of pups in first litter. Thus, indicating that new inbreeding causes more deteriorating effects, whereas old inbreeding causes lesser deteriorating effects and sometimes favorable effects. Using Kalinowski’s [24] approach of new *F*_*K* _ *NEW*_ and old *F*_*K* _ *ANC*_ inbreeding, Mc Parland et al. [44] found a significant unfavorable effect of − 32.4 kg and 3.09 days for MY and AFC, respectively, per 1% increase in *F*_*K* _ *NEW*_. In addition, they observed a significant, but less unfavorable effect of − 8.8 kg and 0.52 day for MY and AFC, respectively, per 1% increase in *F*_*K* _ *ANC*_. In Dutch Holstein-Friesian cattle, Doekes et al. [22] found a significant unfavorable effect of − 2.42 kg for 305-day FY per 1% increase in *F*_*K* _ *NEW*_ and a non-significant, but favorable effect of 0.03 kg for 305-day FY per 1% increase in *F*_*K* _ *ANC*_. Those authors mentioned evidence of purging due to favorable effects found with *F*_*K* _ *ANC*_. In the present study, no significant effects were observed for production traits, however, estimates for *F*_*K* _ *ANC*_ were favorable for MY and PY, whereas *F*_*K* _ *NEW*_ showed unfavorable effects for MY and PY. Conversely, a favorable effect was detected for FY when using estimates from *F*_*K* _ *NEW*_ and *F*_*K* _ *ANC*_. For fertility traits, significant effects were found only for AFS and CTFS. A favorable effect of − 0.77 and − 0.94 days for AFS and CTFS, respectively, per 1% increase in *F*_*K* _ *ANC*_, while an unfavorable effect of 1.58 and 1.00 days for AFS and CTFS, respectively, was found for every 1% increase in *F*_*K* _ *NEW*_. The varying results among these studies could be due to the differences in the populations used, which are subjected to different selection criteria. In the present study, there seems to be no evidence of purging and given the rate at which inbreeding is increasing following the implementation of genomic selection [2], selection will have less time to remove deleterious effects resulting from fast inbreeding [19, 20]. In addition, the evidence of purging due to selection in a controlled or systematic population is being widely debated [24, 46]. Therefore, caution should be taken in concluding that purging has occurred as a result of selection. Furthermore, deleterious alleles could be made less effective by changing environments [24, 47] and the removal of these detrimental alleles are also only partial [48].

## Conclusions

A significant and unfavorable effect of classical inbreeding on all production traits and some fertility traits was found. Genomic inbreeding measures seemed to capture more phenotypic differences between lowly and highly inbred animals. Recent inbreeding was found to show more detrimental effects on both fertility and production traits than ancient inbreeding. However, no substantial evidence of purging was uncovered with ancestral inbreeding. The model-based approach of classifying inbreeding into age classes provided similar results to the pedigree age of inbreeding, hence, in the absence of pedigree records, genomic measures could be used. Overall, heterogeneity of inbreeding depression was observed with recent and ancestral inbreeding. In future studies, the molecular architecture of inbreeding could be investigated to identify regions negatively associated with phenotypic traits.

## Methods

### Pedigree data

Pedigree records for all available animals with genotype and phenotype data were provided by Canadian Dairy Network (Guelph, ON, Canada). The pedigree information consisted of a total of 259,871 individuals that trace back to 1950 as the base year. To ensure that inbreeding estimates were not severely underestimated, pedigree completeness index (PCI) going back five generations and the number of complete generation equivalence (CGE) were estimated using EVA software [49]. Animals with both genotypic and phenotypic data with PCI 0.90 or greater and CGE of 10 or more were retained for further analyses.

### Genotype data

A total of 50,575 genotyped Holstein cows were available with birth year ranging from 1999 to 2017. Cows were genotyped with the Illumina BovineSNP50 Chip (50 K) (Illumina Inc., San Diego, CA) and lower density array panels (10 K - 30 K). Animals with lower density genotypes were imputed to medium density (50 K) using FImpute software [50]. Before editing, SNP information was available for 45,187 SNP markers. For quality control, only autosomal SNP with a call rate > 0.95, minor allele frequency ≥ 0.01 and a difference less than 0.15 between observed and expected heterozygosity frequency were retained for further analyses using SNP1101 [51]. After quality control, a total of 43,126 SNP were retained for further analyses.

### Phenotype data

Phenotypic records of 46,430 cows with first calving date that ranged from 2008 to 2018 were available for production and fertility traits. For production traits, a total number of 21,194 cows had first lactation records on a 305-day basis for milk yield in kg (MY), fat yield in kg (FY) and protein yield in kg (PY). Fertility traits had a total of 52,948 records and these were split up into heifer (first parity) and cow (second parity) traits. Of these records, 33,610 were for heifers and 19,338 were for cows and all animals with cow records also had heifer records. The following fertility traits were considered in this study: age at first service in days (AFS); days from calving to first service (CTFS); number of services (NS); first service non-return rate to 56 days (NRR); days from first service to conception (FSTC). All traits recorded before and during the first parity are termed heifer traits and traits recorded after the first parity were cow traits. NRR was coded as 1 when no subsequent service took place between 15 and 56 days following the first service and coded 0 if otherwise. NS was coded from 1 to 10 and animals with more than 10 services were assigned as 10. AFS was measured in days and considered to be a heifer trait. CTFS was measured in days and considered to be a cow trait. FSTC was measured in days and considered to be both a heifer and cow trait.

### Measures of inbreeding

Pedigree and genomic data were both used in calculating inbreeding coefficients. With pedigree data, inbreeding measures were divided into three categories: 1) classical pedigree inbreeding measure; 2) pedigree age of inbreeding measure and; 3) ancestral pedigree inbreeding measure. For genomic data, inbreeding measures were divided in two categories namely 1) classical genomic inbreeding measure and 2) genomic age of inbreeding measure. A detailed explanation of these categories follows below.

### Pedigree inbreeding measures

Classical inbreeding coefficient (*F*_*PED*_) was estimated for all individuals with phenotypic records by tracing back the pedigree to the founder generation using the algorithm proposed by Meuwissen and Luo [52] as implemented in PEDIG software [53]. Pedigree age of inbreeding coefficient $\left({F}_{PEDn}\right)$ was calculated by tracing back the pedigree *n* generation ago to common ancestors, where *n* represent the specific number of generations to the common ancestors. More specifically, the inbreeding age classes attributable to ancestors from a specific generation is the difference between two successive generations. For example, inbreeding coefficients that occurred due to ancestors in generation seven (*F*_*PED*7 − 6_) can be calculated as the difference between inbreeding coefficients obtained tracing back to seven generations ago *F*_*PED*7_ and coefficients obtained tracing back to six generations ago *F*_*PED*6_ (i.e., *F*_*PED*7 − 6_ = *F*_*PED*7_ − *F*_*PED*6_). This procedure was performed to categorize inbreeding into age classes from most recent to ancient inbreeding. The most recent age traced back was three generations ago (*F*_*PED*3_) because the number of inbred animals in generation two were less than 0.02% of the sample size, while the most ancient age was traced back to generation eight (*F*_*PED*8_) due to having similar inbreeding coefficients with older generations. Pedigree age of inbreeding was estimated using the *vanrad.f* function implemented in PEDIG software [53]. Ancestral pedigree inbreeding was first proposed by Ballou [25] with the concept that alleles with IBD state in an individual have been previously in an IBD state in its ancestor. Kalinowski et al. [24] further modified the ancestral pedigree inbreeding method of Ballou [25] by splitting *F*_*PED*_ into new inbreeding (*F*_*k* _ *NEW*_) and ancient inbreeding (*F*_*k* _ *ANC*_), therefore, *F*_*PED*_ = *F*_*k* _ *NEW*_ + *F*_*k* _ *ANC*_. The difference between *F*_*k* _ *NEW*_ and *F*_*k* _ *ANC*_ is that *F*_*k* _ *NEW*_ is the probability that alleles in IBD state of a given individual is occurring for the first time in the pedigree of the individual, while *F*_*k* _ *ANC*_ is the probability that IBD alleles in an individual have occurred previously in at least one ancestor. Kalinowski ancestral pedigree inbreeding was calculated using a gene dropping approach with 10^6^ replications as implemented in GRAIN [54].

### Genomic inbreeding measures

Two approaches were used in estimating the classical genomic inbreeding: 1) segment-based approach (runs of homozygosity (ROH); *F*_*ROH*_) and 2) marker-by-marker based approach (*F*_*GRM*_). Runs of homozygosity were identified with the deterministic sliding window approach implemented in PLINK using the following criteria: a minimum physical length of 1 Mb; a maximum gap of 500 kb between two successive SNP; a minimum of 20 consecutive homozygous SNP and a minimum density of one SNP per 100 kb. The following formula was used for calculating individual segment based genomic inbreeding:

$$ {F}_{RO{H}_i}=\frac{\sum \limits_{j=1}^n{L}_{RO{H}_j}}{L_{AUTO}} $$

where ${F}_{RO{H}_i}$ is the genomic inbreeding of the *ith* individual, ${L}_{RO{H}_j}$ is the length of the *jth* ROH segment in bp, *n* is the total number of detected ROH and *L*_*AUTO*_ is the total length of the autosomes covered by the SNP in bp.

Inbreeding in the marker-by-marker based approach was calculated by subtracting one from the diagonal of the genomic relationship matrix (***G***) following the proposition of VanRaden [55] and using a 0.5 fixed allele frequency. The formula used in calculating individual marker-by-marker based genomic inbreeding was as follows:

$$ {F}_{GR{M}_i}={\boldsymbol{G}}_{ii}-1 $$

where ${F}_{GR{M}_i}$ is the genomic inbreeding of the *ith* individual and ***G***_*ii*_ is the diagonal element of the genomic relationship matrix.

The genomic age of inbreeding was estimated by classifying already identified ROH length into five different length classes to specify the approximate age or generation in which they occur. As mentioned earlier, deducing the age of inbreeding from ROH length is an expectation that follows an exponential distribution with a mean of 100/2 *g* cM with the assumption that 1 Mb = 1 cM. Therefore, ROH were classified into: 1) 1–2 Mb; 2) 2–4 Mb; 3) 4–8 Mb; 4) 8–16 Mb; and 5) > 16 Mb length classes. These length classes indicate inbreeding resulting from ancient to most recent ancestors. Additionally, genomic age of inbreeding was estimated using the model-based method that uses a Hidden Markov Model (HMM) approach to identify homozygous by descent (HBD) segments [27]. With this method, age of inbreeding is estimated for HBD classes based on a transition probability between different (hidden) HBD segments and non-HBD segments and conditional on the class specificity. The probability of staying in a particular state is calculated as ${e}^{-{R}_k}$, where *R*_*k*_ is the rate specific to the *kth* class. Thus, the length of an HBD segment of any *kth* class is exponentially distributed with rate *R*_*k*_. In the current study, a model with 5 HBD classes was defined following predefined default rates as implemented in the R statistical package “RZooRoH” [27, 42].

### Statistical analyses

To estimate the effect of inbreeding on phenotypes, the same models used in the national genetic evaluation for Canadian Holsteins were adapted from Jamrozik et al. [56], with the inclusion of inbreeding coefficients as a covariate. The specific fixed and random effects fitted for both production and fertility (heifer and cow) traits are presented in Table 5. The fixed effects fitted were as follows: year of calving by season of calving (YSC); age at calving by region of calving (ARC); region by year of birth by season of birth (RYS); month of first insemination (Mf); age at previous calving by month of previous calving by parity (ApMp); age at previous calving by month of first insemination (ApMf). The random effects were: herd by year of birth (HY); herd within RYS (HRYS); service sire by year of insemination (SS); artificial insemination technician (T); animal additive genetic effect (***a***); and error term (***e***). Inbreeding depression was estimated separately for each trait using the following linear mixed model:

1 $$ \boldsymbol{y}=\boldsymbol{Xb}+\beta \boldsymbol{F}+\boldsymbol{Za}+\sum \limits_{\boldsymbol{j}=\mathbf{1}}^{\boldsymbol{n}}{\boldsymbol{W}}_{\boldsymbol{j}}{\boldsymbol{c}}_{\boldsymbol{j}}+\boldsymbol{e} $$

where ***y*** is a vector of phenotypic measurement for MY, FY, PY, AFS, CTFS, NS, NRR and FSTC, ***b*** is a vector of systematic effects, *β* is the coefficient of the linear regression on ***F***, ***F*** is a vector of inbreeding coefficients from pedigree or genomic data (*F*_*PED*_, *F*_*ROH*_ or *F*_*GRM*_), ***a*** is a vector of random additive genetic effects, ***c***_***j***_ is a vector of *jth* non-genetic random effects (HY, HRYS, T and SS) and ***e*** is a vector of random residual effects, n is the number of non-genetic random effects, ***X***, ***Z*** and ***W***_***j***_ are incidence matrices that link the fixed effects, random additive genetic effects and *jth* non-genetic random effects to the phenotypes, respectively. The assumptions for the random effects include: $\boldsymbol{a}\sim N\Big(0,{\boldsymbol{A}\sigma}_a^2$), $HY\sim N\Big(0,{\boldsymbol{I}\sigma}_{HY}^2$), $HRYS\sim N\Big(0,{\boldsymbol{I}\sigma}_{HRYS}^2$), $T\sim N\Big(0,{\boldsymbol{I}\sigma}_T^2$), $SS\sim N\Big(0,{\boldsymbol{I}\sigma}_{SS}^2$) and $\boldsymbol{e}\sim N\Big(0,{\boldsymbol{I}\sigma}_e^2$), where ${\sigma}_a^2$ is the additive genetic variance, ${\sigma}_{HY}^2$ is the herd year variance, ${\sigma}_{HRYS}^2$ is the herd within RYS variance, ${\sigma}_{SS}^2$ is the service sire by year of insemination variance, ${\sigma}_T^2$ is the artificial insemination technician variance, ${\sigma}_e^2$ is the residual variance, ***A*** is the numerator relationship matrix and ***I*** is an identity matrix. For age of inbreeding and ancestral inbreeding, inbreeding depression was estimated using the following linear mixed model:

2 $$ \boldsymbol{y}=\boldsymbol{Xb}+\sum \limits_{\boldsymbol{k}=\mathbf{1}}^{\boldsymbol{m}}{\beta}_k{\boldsymbol{F}}_{\boldsymbol{k}}+\boldsymbol{Za}+\sum \limits_{\boldsymbol{j}=\mathbf{1}}^{\boldsymbol{n}}{\boldsymbol{W}}_{\boldsymbol{j}}{\boldsymbol{c}}_{\boldsymbol{j}}+\boldsymbol{e} $$

where *β*_*k*_ is the coefficient of the linear regression on inbreeding coefficients within the *kth* class of inbreeding (***F***_***k***_), m is the number of inbreeding classes, and all other parameters are the same as mentioned in model I.

**Table 5:** Effects included in the genetic models for genetic parameter estimation for production and fertility traits

Traits^a^	Fixed effects^b^						Random effect^c^				
	YSC	ARC	RYS	Mf	ApMp	ApMf	HY	HRYS	T	SS	A
MY (kg)	**●**	**●**					**●**				**●**
FY (kg)	**●**	**●**					**●**				**●**
PY (kg)	**●**	**●**					**●**				**●**
AFS_H (day)			**●**					**●**			**●**
NS_H			**●**	**●**				**●**			**●**
NRR_H			**●**	**●**				**●**	**●**	**●**	**●**
FSTC_H (day)			**●**	**●**				**●**			
CTFS_C (day)			**●**		**●**			**●**			**●**
NS_C			**●**			**●**		**●**			**●**
NRR_C			**●**			**●**		**●**	**●**	**●**	**●**
FSTC_C (day)			**●**			**●**		**●**			**●**

^a^*MY* milk yield, *FY* fat yield, *PY* protein yield, *AFS_H* age at first service for heifers, *NS_H* number of service for heifers, *NRR_H* 56-day non-return rate for heifers, *FSTC_H* first service to conception for heifers, *CTFS_C* conception to first service for cows, *NS_C* number of service for cows, *NRR_C* 56-day non-return rate for cows, *FSTC_C* first service to conception for cows

^b^*YSC* year of calving by season of calving, *ARC* age at calving by region of calving, *RYS* region by year of birth by season of birth, *Mf* month of first insemination, *ApMf* age at previous calving by month of first insemination by parity, *ApMp* age at previous calving by month of previous calving by parity

^c^*HY* herd by year of birth, *HRYS* herd within RYS, *T* AI technician, *SS* service sire by year of insemination, *A* random animal effect

All linear models in this study were fitted using the restricted maximum likelihood procedure implemented in ASReml 4.1 [57].

## Data Availability

All the necessary information needed to support the results of this paper are included within the article. Data that support the findings of this study are available from Lactanet - Canadian Dairy Network based upon reasonable request.

## Abbreviations

AcMcX: Age at current calving by month of current calving by sex of calf by parity
AFC: Age at first calving
AFS: Age at first insemination
AI: Artificial insemination
ApMf: Age at previous calving by month of first insemination by parity
ApMp: Age at previous calving by month of previous calving by parity
ApMpX: ApMp by sex of calf
ARC: Age at calving by region of calving
bp: Base pair
CDN: Canadian Dairy Network
CGE: Complete generation equivalence
cM: Centimorgan
CTFS: Days from calving to first insemination
F: Inbreeding coefficients
*F*_*GRM*_: Genomic inbreeding estimated from genomic relationship matrix
*F*_*K* _ *ANC*_: Pedigree ancient inbreeding using Kalinowski’s method
*F*_*K* _ *NEW*_: Pedigree new inbreeding using Kalinowski’s method
*F*_*PED*_: Pedigree inbreeding
*F*_*ROH*_: Genomic inbreeding estimated from run of homozygosity
FSTC: Days from first insemination to conception
FY: Fat yield
GRM: Genomic relationship matrix
h^2^: Heritability
HBD: Homozygous by descent
HMM: Hidden Markov model
HRYS: Herd within RYS
HY: Herd by year of birth
IBD: Identity by descent
IBS: Identity by state
IFL: Interval first to last insemination
Kg: Kilogram
Mb: Mega base pair
Mf: Month of first insemination
MfX: Month of first insemination by sex of calf
MY: Milk yield
NRR: 56-day non-return rate
NS: Number of services
PCI: Pedigree completeness index
PY: Protein yield
ROH: Run of homozygosity
RYS: Region by year of birth by season of birth
SS: Service sire
T: AI technician
US: United State
YSC: Year of calving by season of calving
